# Simultaneous assessment of human genome and methylome data in a single experiment using limited deamination of methylated cytosine

**DOI:** 10.1101/gr.278294.123

**Published:** 2024-06

**Authors:** Bo Yan, Duan Wang, Laurence Ettwiller

**Affiliations:** 1 New England Biolabs Incorporated, Ipswich, Massachusetts 01938, USA;; 2 SLC Management, Wellesley Hills, Massachusetts 02481, USA

## Abstract

Multiomics require concerted recording of independent information, ideally from a single experiment. In this study, we introduce RIMS-seq2, a high-throughput technique to simultaneously sequence genomes and overlay methylation information while requiring only a small modification of the experimental protocol for high-throughput DNA sequencing to include a controlled deamination step. Importantly, the rate of deamination of 5-methylcytosine is negligible and thus does not interfere with standard DNA sequencing and data processing. Thus, RIMS-seq2 libraries from whole- or targeted-genome sequencing show the same germline variation calling accuracy and sensitivity compared with standard DNA-seq. Additionally, regional methylation levels provide an accurate map of the human methylome.

Cytosine methylation is the main epigenetic DNA modification found in higher Eukaryotes. In humans, it occurs mainly in a CpG dinucleotide context with the help of DNA methyltransferases (DNMTs), which transfer a methyl group to a cytosine residue to form 5-methylcytosine (m5C) (Moore et al. 2013). Methylation of cytosine is involved in various biological processes, including the regulation of gene expression and chromatin structure (Holliday and Pugh 1975). Additionally, abnormal methylation patterns have been found to play a significant role in disease progression and carcinogenesis (Jones and Baylin 2007). Accordingly, methylation of cytosine can be used as a universal biomarker for the diagnosis of disease, responses to therapeutic interventions, and prognosis (Hulbert et al. 2017; Vrba and Futscher 2018), demonstrating its usage for noninvasive detection of conditions. Furthermore, DNA methylation is a fairly accessible biomarker owing to its low sensitivity to experimental handling.

Techniques for the identification of DNA methylation can be grouped based on the properties used to discriminate between methylated and unmethylated cytosines, namely, enzymatic digestion, affinity enrichment, and enzymatic or chemical conversion. The most commonly used techniques rely on the conversion of cytosine to uracil followed by either hybridization of the converted sequence to methylation arrays or sequencing of the whole-genome (WGBS) or of a reduced representation (RRBS). Converting all cytosines severely reduces sequence complexity, and therefore, these conversion-based techniques have a single aim, namely, to identify methylation. Recently, TET-assisted pyridine borane sequencing (TAPS) (Liu et al. 2019) and DM-seq (Wang et al. 2023) allow the conversion of only methylated cytosine, which significantly improves mapping and coverage.

A direct dual readout of both the sequence and methylation information on the same DNA molecule can be achieved using single-molecule sequencing platforms, such as Oxford Nanopore Technologies (Rand et al. 2017; Simpson et al. 2017) or Pacific Biosciences (PacBio) (Clark et al. 2013). Oxford Nanopore Technologies uses changes in the pore ionic current signal to detect DNA modifications, whereas PacBio relies on changes of the polymerase speed between the fluorescent pulses to indicate modification (Clark et al. 2012). In both instances, these technologies require the sequencing of the original DNA molecule to preserve the associated methylation information.

Such a readout of both sequence and methylation information cannot be directly obtained from short-read sequencing because amplification of the original DNA molecule is necessary for clustering. We and others have therefore used the redundancy of the double-stranded DNA to identify DNA methylation information and genomic variants simultaneously (Laird et al. 2004; Liang et al. 2021; Yan et al. 2022; Füllgrabe et al. 2023). Although this setup allows for dual readouts in a single data set, the experimental application of these techniques proved to be a significant departure from standard library preparation as the procedure involves linking double-stranded DNA together.

Chemical or fortuitous deamination can also be used as means to identify cytosine methylation. For example, Gokhman et al. (2014) elegantly harness DNA damage resulting from the natural degradation processes of inappropriately stored DNA to identify methylated and unmethylated cytosines in ancient DNA. Similarly, we developed RIMS-seq, a new method to identify methylase specificity in bacteria (Baum et al. 2021). To perform RIMS-seq, genomic DNA is subjected to a limited heat alkaline treatment step that induces a deamination of a fraction of m5C. Although unmethylated cytosines are also deaminated, they are effectively eliminated during the amplification step owing to the usage of a proofreading polymerase that stalls at dU sites. Thus, only the deaminated m5C results in a C-to-T transition in sequencing reads. m5C sites are therefore identified by virtue of their elevated C-to-T transition rate. Importantly, the protocol requires only a minimal departure from standard DNA-seq, and although the deamination is large enough to slightly elevate the C-to-T error rate at m5C sites, this level of deamination does not affect library yields and sequencing quality (Baum et al. 2021).

In this study, we developed RIMS-seq2 for the identification of methylated loci in humans. We first adapted the experimental protocol to assess concerted methylation at nearby CpG sites and apply RIMS-seq2 on well-known cell lines as well as matched tumor/normal tissue samples. We demonstrate broad applicability of this technology in the simultaneous identification of sequence and methylation in a single experiment with minimal modification of the standard library protocol and sequencing qualities matching DNA-seq for variant calling.

## Results

### Considerations for the application of RIMS-seq2 to human genome sequencing

A previous version of the RIMS-seq protocol has been used for the identification of methylase specificity in bacterial genomes using an overall elevation of C-to-T transition rate of ∼0.1% (Baum et al. 2021). This deamination level is generally enough for the identification of sequence context(s) surrounding the methylated cytosine characteristic of the prokaryotic methylase specificity(ies). To apply RIMS-seq2 for the identification of human methylation, both the experimental and analytical protocols need to be adapted to the fact that methylation happens almost exclusively at CpG sites and only a subset of these sites are methylated. Human genomic DNA also harbors m5C as well 5-hydroxymethylated cytosines (5hmC). Using genomic DNA of a T4gt bacteriophage that contains 5hmC (Miller et al. 2003), we showed that the heat alkaline deamination of 5hmC, resulting in 5hmU, exhibits a conversion rate similar to that of m5C in a RIMS-seq2 protocol (Supplemental Fig. S1A). Furthermore, the conversion rate at 5hmC is consistently observed at ∼1% in all sequence contexts examined (Supplemental Fig. S1B). These results demonstrate that RIMS-seq, analogous to bisulfite sequencing and EM-seq, indiscriminately identifies m5C + 5hmC.

Experimentally, we elevated the pH (1 M NaOH) and tuned the deamination duration to 30 min at 60°C, achieving a ∼1% C-to-T transition at m5C sites. Increasing the C-to-T transition rate at m5C reduces the number of sites needed to estimate the methylation levels. Additionally, we have implemented a modification to the previously published protocol by incorporating uracil DNA glycosylase (Lindahl et al. 1977) for uracil base excision before amplification of the library. This refinement serves to further diminish the background noise associated with C-to-T transitions at unmethylated sites, thereby enhancing the signal-to-noise ratio (Methods). Under these conditions, the C-to-T transition is expected to increase to about 1000-fold at m5C sites compared with the background error rate of Illumina. This fold increase represents the “goldilocks” zone to identify methylation in human without affecting sequencing quality for a variety of standard genomic applications. For example, we expect the identification of germline variations to be done using standard tools for variant calling such as GATK (McKenna et al. 2010) without affecting call accuracy.

Nevertheless, at current standard sequencing depth (30-fold coverage or above), this level of deamination does not provide base-resolution methylation identification but rather provides regional aggregated methylation levels (RAMLs) over a defined genomic region. We estimated that the accurate evaluation of the methylation status can be safely done at about 100 combined CpG sites or above. Considering a combined 100 CpG sites and a 1% deamination rate at methylated CpG sites, a 30-fold read coverage at these sites would result in an average of 30 C-to-T deamination events. This number of CpG sites represent about or less than the average size of a single CpG island (CGI), a resolution that is compatible with most epigenetic applications. Indeed, functional genomic regions ranging between a few hundred and a few thousand bases tend to be regionally methylated or unmethylated in concert (Chen et al. 2016), and a number of established protocols for methylation analysis have already been taking advantage of this concerted signal to identify such local aggregate as opposed to base-resolution methylation levels (Weber et al. 2005; Brinkman et al. 2010).

### Whole-genome and targeted-genome sequencing

To demonstrate the applicability of RIMS-seq2 in the simultaneous sequencing of DNA and methylome, we performed RIMS-seq2 on human genomic DNA. RIMS-seq2 whole-genome sequencing (WGS) was performed on NA12878 genomic DNA. Exome target capture (TES) using RIMS-seq2 was performed on both NA12878 and K562 genomic DNA as well as genomic DNA extracted from frozen/tumor breast tissue. Finally, to ensure reproducibility and compatibility with DNA-seq, we performed technical replicates on the target captures and included a control DNA-seq using the same source of starting material, respectively.

For WGS, ∼150 ng of NA12878 genomic DNA was used to generate 4.6 billion paired-end reads, achieving an average coverage >200×. For exome sequencing, we used 50–100 ng of genomic DNA to generate about 100–200 million paired-end reads, achieving an average of more than 40-fold coverage (Supplemental Table S1).

Reads were trimmed and mapped to the human genome (GRCh38) using Bowtie 2 (Langmead and Salzberg 2012). C-to-T transitions at CpG sites were identified for each individual read and combined over predefined genomic regions to obtain C-to-T transition rates (Methods) (Supplemental Table S1). Transition rates were subsequently calibrated to obtain overall methylation levels in these genomic regions (see below).

#### RIMS-seq2 shows a linear relationship between transition rates and methylation levels

We first evaluated how the C-to-T transition rate of RIMS-seq2 correlates with local methylation level in CGIs, promoters, or exonic regions. For this, we defined methylation levels across the NA12878 genome using published gold-standard data sets. More specifically, we calculated the weighted average methylation from three data sets derived from whole-genome bisulfite sequencing (WGBS), EM-seq (Vaisvila et al. 2021), and Nanopore (Jain et al. 2018) done on NA12878 (Methods). Because these data sets result from independent technologies for methylation identification, the weighted average methylation should minimize the bias inherent to each method (Olova et al. 2018) and provide closer to “true” methylation levels (see Methods). Next, CGIs, promoters, or exonic regions with similar methylation levels were binned together, and the excess of C-to-T transition rate observed in RIMS-seq2 is computed for each bin.

**Figure 1.**
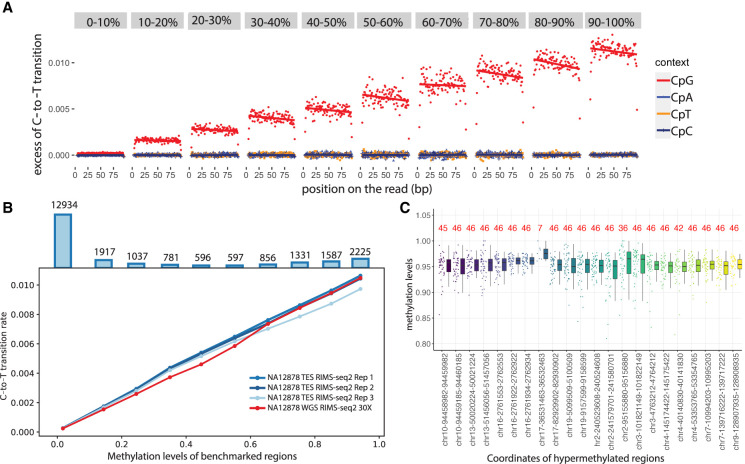
Linear relationship between transition rates and methylation levels. (*A*) Whole-genome RIMS-seq2 excess of C-to-T transition rates in read 1 compared with read 2 (imbalance) function of the position on the read (in base pairs). CGI regions are binned into 1%–10% to 90%–100% methylation levels. The rate of C-to-T transition in each bin was computed for CpG (red), CpA (blue), CpT (orange), and CpC (purple) contexts. (*B*) The rate of C-to-T transition and benchmarked methylation in binned CGIs fits a positive linear regression model for all NA12878 RIMS-seq2 whole-genome sequencing (red) and exome sequencing in triplicates (blue). *Top* bar plots represent the total number of CGI in each bin. (*C*) Twenty-two stably methylated regions across a broad range of tissues and cell types used as internal control for methylation level calibration. The number in red indicates the number of available human WGBS samples used for methylation analysis. Genomic regions coordinates are derived from the human genome (GRCh38).

As expected, we found the excess of C-to-T transition to be correlated with methylation levels in CGIs (Fig. 1A), promoters, and exonic regions (Supplemental Fig. S1C,D). Such correlation was only observed in a CpG context; the other contexts do not show an excess of C-to-T transition, consistent with the fact that the vast majority of methylation events in human happen in CpG context (Fig. 1A).

To demonstrate linearity between methylation levels and RIMS-seq2 C-to-T transition rates, we performed a regression analysis test for the binned CGIs (Fig. 1B,C), promoters, and exons (Supplemental Fig. S1E,F) for both whole-genome and targeted RIMS-seq2. The resulting regression analysis reveals positive linear relationships between transition rates and the methylation levels in all regions for both whole-genome (*R*^2^ = 0.99, *P-*value of variable < 0.01, coefficient = 0.01) and targeted RIMS-seq2 (*R*^2^ = 0.99, *P-*value of variable < 0.01, coefficient = 0.01) (Supplemental Table S2). This result indicates that quantitative measurement of methylation level using RIMS-seq2 is achievable and that the absolute quantification can be calibrated using only two data points.

#### Development of a linear model between transition rates and methylation levels

Although the heat alkaline deamination treatment is aiming at ∼1% C-to-T transition at m5C sites, experimental variations, genomic DNA quality, and sequencing runs may affect the C-to-T transition rate at both C and m5C for each individual experiment. It is therefore important to perform a calibration specific to each sequencing run. Having established the linear correlation between transition rates and methylation levels, this calibration can be done using two sets of internal controls only representing hypermethylated regions and unmethylated sites, respectively.

For hypermethylated regions, a set of 24 internal control regions was selected as stably hypermethylated across a broad range of tissues and cell types (Edgar et al. 2014) including in NA12878 (Fig. 1C; Supplemental Table S3). We used the non-CpG sites in these regions to estimate the C-to-T transition rate at unmethylated cytosines. Applied to all the RIMS-seq2 data sets obtained for this study (Supplemental Table S1), we obtained the C-to-T transition rate at m5C to be ∼1% for m5C and 10 × 10%–5% for C, which is a 1000-fold increase in C-to-T transition rate at methylated compared with unmethylated sites.

Next, using these stably hypermethylated regions, we explored the impact of sequence context on calibration, notably the upstream base relative to the deaminated base. The effect of the upstream base to CpG sites is subtle but not negligible with sample and run variations (Supplemental Fig. S2A). The ApCpG context for which an adenosine is found upstream of the CpG repeatedly shows the lowest C-to-T transition (Supplemental Fig. S2A). The sequence context is therefore incorporated into the calibration procedure as a parameter to improve methylation call accuracy. We also observed an effect of the sequencing cycles, notably for the two first and two last cycles of paired-end sequencing (Supplemental Fig. S2B). For these cycles, the C-to-T transition is lower than expected for fully methylated sites and removed from the methylation analysis.

In addition, because the deamination rate only moderately increases the signal over the noise ratio, RIMS-seq2 is attuned to variations from the reference genome. Thus, we also investigated the effect of single-nucleotide polymorphism (SNP) on methylation calls. We observed an increase in methylation call accuracy if the publicly available NA12878 SNP positions were to be removed prior to methylation call (Supplemental Fig. S2C). An equivalent improvement was obtained if the SNP positions were identified directly from the RIMS-seq2 data sets and subsequently used for methylation call (for details, see below) (Supplemental Fig. S2C). This result demonstrates that an external SNP data set is not required for this analysis. Thus, prior to calibration, the positions identified by RIMS-seq2 as SNPs were removed from the methylation calls. Finally, we assessed the influence of the mapping quality metric MAPQ on methylation calls and found that for Bowtie 2 mapping, a MAPQ of 10 yielded the highest accuracy (Supplemental Fig. S2D).

### Methylation calling at regional resolution

**Figure 2.**
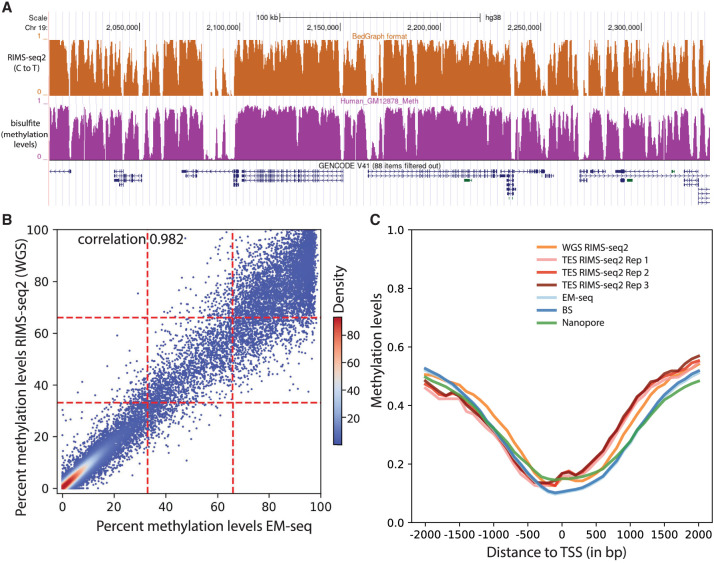
Performance of RIMS-seq2 (methylation). (*A*) C-to-T profile at a specific locus (combined 30 CpG sites) compared to methylation profile from bisulfite sequencing. (*B*) Methylation-level (RAML) correlation at CGIs between RIMS-seq2 and EM-seq. Each point corresponds to a CGI region (raw data provided in Supplemental Table S4). The plotting area has been divided into nine quadrants. (*C*) Methylation profiles at promoters measured by RIMS-seq2 (both WGS and TES), EM-seq (light blue), bisulfite sequencing (dark blue), and Nanopore (green). The overall percentage methylation of CpG sites was measured using 100 bp sliding windows within 2 kb upstream of and downstream from transcription start sites (TSSs). TSSs were defined using UCSC annotation. Distance to a TSS is measured in base pairs.

We are now addressing the ability of RIMS-seq2 to define methylation at regional resolution. As a first pass, profiles of C-to-T rate were compared with a published WGBS methylation profile performed on NA12878. Visual inspection of the methylation profile indicates that the C-to-T profile correlates closely with the methylation-level profiles from WGBS sequencing (Fig. 2A).

To quantify how correlated the methylation levels are between RIMS-seq2 and other technologies for methylation analysis such as bisulfite sequencing or EM-seq, as well as technologies that provide both sequence and methylation readouts such as Nanopore, 5-letter-seq (Füllgrabe et al. 2023), and Methyl-SNP-seq (Yan et al. 2022), we proceed with a genome-wide comparison of various publicly available methylation data sets done on the same cell lines. Because RIMS-seq2 cannot reliably identify methylation at base resolution with current standard sequencing depth, we aim at obtaining RAML values at defined genomic regions. For this, we delineated genomic regions of interest for methylation identification such as CGIs, promoters, and exonic regions and performed local calibrations using the linear model described above for the combined CpG sites in these regions. For comparison, we also performed similar regional methylation aggregates with the public methylation data sets. We found that the large majority of CGIs (66%) and promoters (71%) have methylation levels <30% (Fig. 2C), indicating hypomethylation in these regions consistent with the fact that these regions tend to be hypomethylated (Weber et al. 2007). Conversely, only 30% of exonic regions are hypomethylated.

For comparison with existing technologies, we computed standard correlation coefficients and complemented the correlation coefficients with a measure of quadrant consistency for an additional metric of similarity. Technical replicate analysis of RIMS-seq2 performed on the same sample shows good methylation concordance with a quadrant consistency of 91% and a 0.95 correlation (Supplemental Fig. S3). The same correlation levels are observed between triplicate RIMS-seq2 exome sequencing and WGS (Supplemental Fig. S3). High correlation levels are observed in replicates of both cell lines as well as the frozen tissue (Supplemental Figs. S3, S4). The correlations between RIMS-seq2 and other technologies are similarly high, ranging from 0.94 to 0.98 (Fig. 2B; Supplemental Fig. S4). The agreement between RIMS-seq2 and other methods that allow DNA sequencing and methylation calling is even higher, with correlations of 0.982 and 0.978 between RIMS-seq2 and 5-letter-seq (Füllgrabe et al. 2023) and methyl-SNP-seq (Yan et al. 2022; Füllgrabe et al. 2023). Comparison between Nanopore WGS and RIMS-seq2 exome sequencing revealed a strong concordance between data sets with 0.945 correlation (Supplemental Fig. S4).

### DMR identification using RIMS-seq2

We applied RIMS-seq2 to the detection of differentially methylated regions (DMRs) between matched paired frozen breast tissue and tumor samples. Exome sequencing was performed in triplicates and compared with EM-seq performed in duplicates on the same samples (Methods). Analysis of the frozen tissue samples revealed a noteworthy methylation correlation, with an average of 0.95, observed between RIMS-seq2 and EM-seq (see Supplemental Fig. S4C). This result supports the versatility of RIMS-seq2 in handling more complex samples.

**Figure 3.**
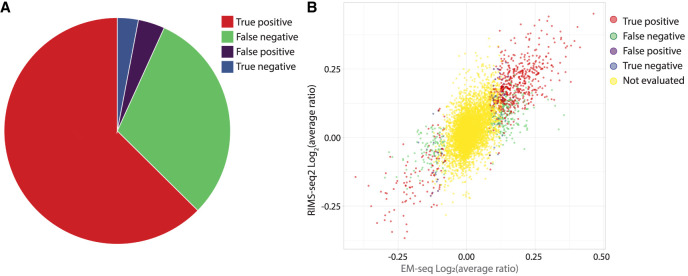
DMR identification using RIMS-seq2. (*A*) Pie chart of the performance of RIMS-seq2 compared with EM-seq with the number of CpG islands (CGIs) that are found as true positive (red), false negative (green), false positive (purple), and true negative (blue). (*B*) Correlation between the differential methylation in EM-seq and RIMS-seq2. The “not evaluated” (yellow) category represents CGIs with differences <10%, which were not used for analysis.

Next, we assessed the ability of RIMS-seq2 to identify differentially methylated CGIs that also show a significant difference in methylation levels between tissue and tumor according to EM-seq (Methods) (Supplemental Table S5). Analysis shows that out of the 932 EM-seq differentially methylated CGIs, 626 also exhibited differential methylation in RIMS-seq2 (Fig. 3A, true positive). Notably, the majority of RIMS-seq2 false negatives were identified in DMRs that, according to EM-seq, displayed intermediate methylation differences between cancer and tissue (see Fig. 3B). This suggests that RIMS-seq2 may have lower sensitivity to intermediate differences in methylation levels but still maintain accuracy for more substantial methylation changes.

### Comparison with available technologies for genome sequencing

#### Coverage bias, insert sizes, chimeras, and on-target sequencing

We perform basic quality control on both the RIMS-seq2 data set and standard DNA sequencing (Supplemental Fig. S5). Damage to the DNA has been shown to reduce the amplicon size after library preparation (only small fragments are amplified in severely damaged DNA) (Pääbo 1989). We therefore sought to compare the insert size distribution between RIMS-seq2 and DNA-seq to assess the damaging impact of the heat alkaline treatment. The distribution of both RIMS-seq2 and DNA-seq insert sizes is similar (Supplemental Fig. S5A), indicating that the heat deamination treatment step did not have a significant impact on amplicon sizes.

#### Germline variant calling

**Figure 4.**
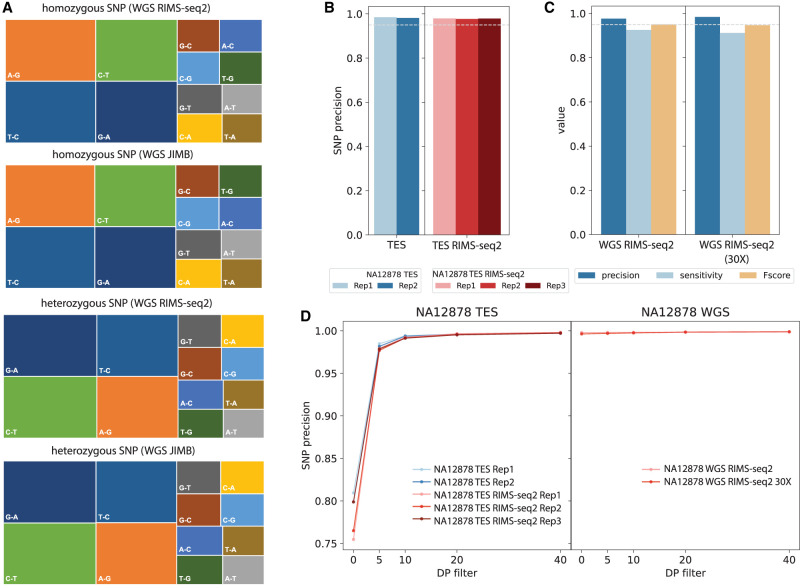
Performance of RIMS-seq2 (DNA sequencing). (*A*) Germline transitions/transversion profiles for JIMB and RIMS-seq2 WGS for homozygous (*top*) and heterozygous (*bottom*) SNPs. In all cases, transitions represent the majority of the SNPs identified. (*B*) Precision of variant calling for standard DNA-seq and RIMS-seq2 targeted-exome sequencing. (*C*) Precision, sensitivity, and *F*-score for WGS RIMS-seq2 (full data set and downsampled to 30-fold coverage). (*D*) The effect of the DP filter on precision.

The deamination conditions should not interfere with genome sequencing for a variety of applications such as the identification of germline variations. To demonstrate that RIMS-seq2 accurately identifies germline variation, we use the GATK pipeline (McKenna et al. 2010) for variant calling on both the whole-genome and exome RIMS-seq2 data and compare the results to JIMB variants focusing on SNPs. If deamination is interfering with variant calling, the overall fraction of C-to-T or G-to-A transition should be higher in RIMS-seq2 data sets. Nonetheless, the profile of SNPs closely resembles the JIMB SNPs, indicating that the overall SNP profiles are not affected (Fig. 4A). The metrics of precision, sensitivity, and *F*-score of the SNPs called using RIMS-seq2 data are similar to standard DNA sequencing (Fig. 4B–D).

## Discussion

RIMS-seq2 enables the simultaneous identification of sequence and methylation for short-read sequencing. Importantly, the experimental setup closely resembles a standard library preparation with minimal changes, and the resulting data can be analyzed using standard variant calling. These features make this technology an extremely easy to deploy strategy for simultaneous germline variation and methylation identification in large-scale sequencing laboratories.

Simultaneous germline variation and methylation identification are now routinely performed using long-read sequencing (Rand et al. 2017; Simpson et al. 2017) in the context of native DNA for which no prior amplification has been performed, limiting the range of applications to direct methylation sequencing. Comparison between Nanopore WGS and RIMS-seq2 exome and genome sequencing revealed a strong concordance between data sets. RIMS-seq2 can be performed with or without amplification and thus can be performed on a greater set of applications.

At current standard sequencing depth of 30-fold for WGS, RIMS-seq2 cannot achieve base resolution. Moreover, because of the minimal deamination rate (1%) of methylated cytosines and subsequent calibration to determine percentage methylation, regional deviations from the average 1% deamination rate are magnified. This amplification contributes to increased variation in methylation percentages, particularly prominent in fully methylated regions. Nonetheless, this resolution is sufficient to tightly correlate at a regional level with established technologies and identify DMRs. To increase coverage and resolution, target enrichment followed by RIMS-seq2 allows for the sequencing of panels of regions such as exome sequencing. The novelty about target enrichment using RIMS-seq2 is the ability to call methylation using standard probes (four bases). The method is as expensive as performing targeted DNA-seq at equivalent coverage. As exemplified with exome sequencing, RIMS-seq2 is expected to perform well with any commercially available target enrichment panels or custom panels that target genomic regions in humans and other organisms.

With constant increase in sequencing throughput and price drops, it is conceivable that several-thousand-fold coverage of the human genome can be achievable in a routine fashion. Such coverage levels are already possible for targeted-genome sequencing, enabling base-resolution methylation calls using RIMS-seq2.

Importantly, RIMS-seq2 comes at almost no extra cost compared with a standard DNA-seq and is compatible with a large number of presequencing treatments such as target enrichment, as demonstrated in this study, but also chromatin accessibility sequencing, ChIP-seq, or single cell. In conjunction with target enrichment, quality-control metrics show essentially identical performance compared with the DNA-seq.

As we have demonstrated in this study, the 1% deamination at CpG sites does not interfere with germline variation calls because variant frequencies are significantly above the deamination rates. Nonetheless, RIMS-seq2 may interfere with the identification of rare somatic mutations for which the frequency of variant is similar to the deamination rate. In these cases, deamination may confound the identification of rare somatic mutations and may not be used for these applications. Alternatively, algorithms for somatic mutations can be adapted to distinguish true mutations from limited deamination.

We have shown that the sequencing context has a minimal effect on the deamination rate of methylated cytosine. Thus, RIMS-seq2 can be directly applied to the identification of methylation in organisms that methylate cytosines in other contexts than CpG context such as plants and prokaryotes. Likewise, in this work, we choose to focus our analysis on CpG sites, but CpA sites can be included for samples that are known to have significant levels of CpA methylation.

## Methods

### RIMS-seq2 library preparation

We used human genomic DNA isolated from GM12878 cells (referred as NA12878, provided by Coriell Institute), K562 cells (provided by ATCC), and paired breast tumor/tissue (Biochain D8235086-PP-10) for RIMS-seq2 sequencing in this study. We used 100–200 ng and 50–100 ng of genomic DNA for RIMS-seq2 WGS and TES library preparation, respectively. We performed RIMS-seq2 following the published protocol (Baum et al. 2021) with some modifications. We used the NEBNext Ultra II library prep kit (NEB E7645) following the manufacturer's instructions until the USER treatment step included following the adapter ligation step. After this first USER treatment step, the sample was subjected to heat alkaline deamination in 1 M NaOH (final concentration) for 30 min at 60°C. The sample was subsequently cooled down on ice, and equal moles of acetic acid were added to a final concentration of 1 M to neutralize the pH. DNA was purified using a Zymo oligo clean and concentrator kit (D4060 Zymo Research) following the protocol for clean-up of DNA >80 nt. An additional USER treatment step was performed to the purified DNA by adding 2 μL USER (included in all the NEB index primer kits) and incubating for 15 min at 37°C. Finally, we used the USER-treated DNA as a template for PCR amplification using NEBNext Ultra II Q5 master mix. Eight samples were amplified and pooled for target enrichment using the Twist comprehensive exome panel (Twist 102031), following the manufacturer's recommendations. The enriched DNA was subsequently amplified with NEBNext Ultra II Q5 master mix, and both the whole-genome and targeted libraries were sequenced on the Illumina NovaSeq 6000 platform using a paired-end mode with a read length of 100 bp.

### EM-seq library preparation

Fifty nanograms of genomic DNA from breast tumor/tissue was used to prepare EM-seq libraries as per the manufacturer's instructions (NEB E7120). The Illumina NovaSeq 6000 sequencer was used to sequence the libraries in a paired-end mode, generating 100 bp reads. The evaluation of EM-seq conversion efficiency (>99.7%) was performed by utilizing unmethylated lambda genomic DNA as a spike-in.

### Reference genome and other annotation files

We used the GRCh38 human reference genome (hg38), UCSC human CGI annotation, and known human SNP files used for GATK base quality recalibration as previously explained (Yan et al. 2022).

### RIMS-seq2 data processing

Initially, Trim Galore! (version 0.6.4; https://github.com/FelixKrueger/TrimGalore) was utilized to trim the Illumina adapter from the reads. Additionally, for NA12878 WGS RIMS-seq2, the first two bases of Read1 were trimmed owing to their poor quality (‐‐clip_R1 2). Next, the trimmed reads were aligned to the hg38 human reference genome using Bowtie 2 (version 2.3.0) with the default parameters for paired-end mapping and inclusion of the read group identifier defined by @RG. To ensure the accuracy of downstream analysis, we discarded improperly mapped reads using SAMtools (version 1.14) (Li et al. 2009) and PCR duplicates using Picard tools (version 2.26.11) (https://broadinstitute.github.io/picard/) MarkDuplicates.

### RIMS-seq2 C-to-T transition counting

To prevent the repetitive counting of the same transition event, we utilized a custom script (TrimOverlappingReadPair.py) to remove the overlapping regions between Read1 and Read2 from Read2. Next, we separated the mapped Read1 (-f 64) and Read2 (-F 64) using SAMtools. Then we compared the Read1 and Read2 mapping to the hg38 genome using SAMtools mpileup with the following parameters: ‐‐min-MQ 10 ‐‐min-BQ 30 ‐‐output-BP-5 ‐‐no-output-ins ‐‐no-output-ins ‐‐no-output-del ‐‐no-output-del ‐‐no-output-ends. The C-to-T transition at CpG sites was then counted for both Read1 and Read2 in a context-dependent manner using a custom script (CountErrorMpileup.py), with the following parameters: ‐‐REF C ‐‐BASE T ‐‐left 1 ‐‐right 0 for Read1 and ‐‐REF G ‐‐BASE A ‐‐left 0 ‐‐right 1 for Read2. Context-independent counting was performed using ‐‐left 0 ‐‐right 0 for both Read1 and Read2. Furthermore, the removal of SNP positions and specific sequencing cycles from counting was accomplished using the ‐‐vcf and ‐‐cycle options, respectively, to enhance the accuracy of downstream methylation prediction. Finally, we added the C-to-T transition of all the CpG sites in the targeted region(s) such as CGI using a custom script (CountErrorRegion.py). These regional C-to-T transition counts (defined as *Error*) and all cytosine counts (C + C to T, defined as *Total*) were used for regression analysis and methylation prediction. The C-to-T transition rate (R) equals to error divided by total.

### Data processing and methylation quantification by other methods

We downloaded the previous published 5-letter-seq (Füllgrabe et al. 2023) methylation information from the NCBI Gene Expression Omnibus (GEO; https://www.ncbi.nlm.nih.gov/geo/) under accession number GSE208549 and Methyl-SNP-seq (Yan et al. 2022) methylation information from GEO under accession number GSE206253. The EM-seq or WGBS data set were downloaded from NA12878 ENCODE WGBS (ENCODE: ENCSR890UQO), NA12878 NEB WGBS (NCBI Sequence Read Archive [SRA; https://www.ncbi.nlm.nih.gov/sra]: SRR10532136, SRR10532135, SRR10532127, and SRR10532126) (Vaisvila et al. 2021), NA12878 NEB EM-seq (NCBI SRA: SRR10532145, SRR10532144, SRR10532139, and SRR10532138) (Vaisvila et al. 2021), and K562 ENCODE WGBS (ENCODE: ENCSR765JPC). The EM-seq of breast tumor/tissue was generated in this study as mentioned above.

We processed these data sets and extracted methylation information using the Bismark pipeline: (1) For a fair comparison, we shortened the paired-end reads to 100 bp long and trimmed the Illumina adapters as well as the first two bases of Read2 (Trim Galore! ‐‐clip_R2 2); (2) we aligned trimmed reads to the human GRCh38 genome using Bismark (version 0.22.3); (3) we filtered the PCR duplicates and incomplete bisulfite conversion using Bismark deduplicate_bismark and filter_non_conversion, respectively; and (4) we combined the replicates and extracted CpG site methylation using bismark_methylation_extractor. The methylation level of targeted regions such as CGI or promoters was calculated as explained previously (Yan et al. 2022).

For NA12878 BS-tagging sequencing (mentioned as Tn5 BS in Supplemental Fig. S4A) (Suzuki et al. 2018), we converted its processed methylation information on hg19 to hg38 using the UCSC liftOver tool (Hinrichs et al. 2006). For NA12878 Nanopore sequencing, the data processing and methylation quantification were described previously (Yan et al. 2022).

### Regression analysis between the C-to-T transition rate and the methylation level

We mathematically described the relationship between the RIMS-seq2 C-to-T transition rate and the methylation level of CpG sites in certain regions of the GM12878 cells. We chose the CGIs, promoter regions that are defined as 1000 bp upstream of and 100 bp downstream from the annotated TSS, and Twist target enrichment exome bait regions for this analysis. The methylation level of these regions was measured by three methods including WGBS, EM-seq, and Nanopore sequencing as mentioned above. We used regions having coverage of CpG sites of 50 or more in WGBS and EM-seq and of 20 or more in Nanopore for analysis.

To reduce method bias, we integrated the measurements from these three methods, which is defined as the benchmarked methylation level for the region *n* (*BM*_*n*_), using the following steps: First, we calculated the proportion (*P*_*n*,*i*_) and weight (*W*_*n*,*i*_) of each method for region *n* given by

$$P_{n,i}=\frac{C_{n,i}}{\sum_{m=1}^{N}C_{m,i}},$$

$$W_{n,i}=\frac{P_{n,i}}{\sum_{\;j=1}^{3}P_{n,j}},$$

where *i*, *j* ∈ {1, 2, 3} denotes the three methods, *n*, *m* ∈ {1, 2, …, *N*} denotes the region, and *C*_*n*,*i*_ represents the coverage of CpG sites by the method in certain region *n*. Second, for region *n*, we selected the two measurements with closest values given by

$$({\;\hat{i}}_{n},\;{\hat{j}}_{n})=\arg\underset{i,j}{\min}|M_{n,i}-M_{n,j}|,$$

where *M*_*n*,*i*_ and *M*_*n*,*j*_ stand for the methylation level measured by method *i* and *j* for region *n*. We computed the *BM*_*n*_, which is the weighted average methylation by

$$BM_{n}=\frac{W_{n,\;{\hat{i}}_{n}}M_{n,\;{\hat{i}}_{n}}+W_{n,\;{\hat{j}}_{n}}M_{n,\;{\hat{j}}_{n}}}{W_{n,\;{\hat{i}}_{n}}+W_{n,\;{\hat{j}}_{n}}}.$$

Next, we classified these regions into *N*_*Bin*_ bins (*N*_*Bin*_ = 10) with equal width based on the benchmarked methylation level. The lower bound (*LB*_*k*_) and upper bound (*UB*_*k*_) of each bin *k* is defined as

$$LB_{k}=\frac{k-1}{N_{Bin}},$$

$$UB_{k}=\frac{k}{N_{Bin}},$$

with *k* ∈ {1, 2, …, 10}. The average methylation level of bin *k* (*ABM*_*k*_) is calculated by

$$ABM_{k}=\frac{\sum_{n=1}^{N}BM_{n}1_{LB_{k}<BM_{n}<UB_{k}}}{N},$$

where *N* is the number of CGI regions in bin *k*, and “**1**” represents the indicator function. For a given condition,

$$1_{cond}=\{\begin{array}{cc} 0,\; & cond=FALSE\;\\ 1,\; & cond=TRUE.\; \end{array}$$

Therefore, $BM_{n}1_{LB_{k}<BM_{n}<UB_{k}}$ means the benchmarked methylation within the lower and upper bound.

We also measured the RIMS-seq2 C-to-T transition rate with the following parameters as explained above: ‐‐min-MQ 10 for SAMtools mipleup; ‐‐vcf using SNP annotation based on JIMB WGS of NA12878 for counting error using CountErrorMpileup.py. Then the total counts (*Total*_*n*_) and C-to-T transition counts (*Error*_*n*_) of the CpG sites in all the regions included in bin *k* were added together to represent the transition rate (*R*_*k*_) of this bin:

$$R_{k}=\frac{\sum_{n=1}^{N}Error_{n}}{\sum_{n=1}^{N}Total_{n}},$$

with *N* as the number of regions in bin *k*.

Finally, we performed the regression analysis to evaluate the linear relationship between the methylation level *ABM*_*k*_ and transition rate *R*_*k*_. By interpreting the *P*-value of variable and intercept, we concluded that these two variables fit the linear model as shown in Supplemental Figure S1, E and F:

$$R_{k}=\alpha +\beta \cdot ABM_{k}+\varepsilon_{k}.$$

### RIMS-seq2 RAML quantification

Given the methylation level and C-to-T transition rate fitting a linear model, we can predict the RAML based on the C-to-T transition rate of the target region. We used 24 hypermethylated regions (Supplemental Table S3) for which the CpG sites are known and confirmed to be stably methylated in the human genome to establish the linear model. We use *R*_*0*_ and *R*_*100*_ to represent the C-to-T transition rate of the non-CpG cytosines and the CpG sites in these stably hypermethylated regions, respectively. It is worth mentioning that we adjusted R_100_ corresponding to the real methylation level in these regions based on all the published human WGBS data from ENCODE as annotated in the Supplemental Code, given by

$$WM=\frac{\sum_{n=1}^{N}M_{n\;}}{N},$$

$$R_{100}=\frac{\sum_{n=1}^{24}\frac{Error_{n}}{WM_{n}}}{\sum_{n=1}^{24}Total_{n}},$$

where *M*_*n*_ represents the methylation level of a certain hypermethylated region based on one human ENCODE WGBS data set, and *N* is the number of available human WGBS data sets for this region. Therefore, *WM* represents the mean methylation level of this hypermethylated region in human genome.

The RAML can be estimated by

$$\frac{Error-Total\;*\;R_{0}}{(R_{100}-R_{0})\;*\;Toal}.$$

*Total* and *Error* are the cytosine counts and C-to-T transition counts in the corresponding region as explained above. Because the methylation level needs to be between 0% and 100%, we apply a lower and upper bound for the estimated methylation level (*Meth*):

$$Meth=\min\left(1,\;\max\left(0,\frac{Error-Total*R_{0}}{(R_{100}-R_{0})*Total}\right)\right).$$

### DMR analysis

We examined whether there was a significant difference in the methylation levels of specific CGIs, with a threshold set at a ≥10% difference between breast tumor and paired nontumor breast tissue.

First, we utilized TES RIMS-seq2 for three replicates of both breast tumor and paired nontumor breast tissue. We analyzed the RIMS-seq2 results to determine the significance of methylation level variations exceeding 10% between tumor and tissue samples. The detailed procedure is outlined below.

For a specific CGI region *n*, we identified the two measurements with the closest values from the methylation results obtained from the three RIMS-seq2 replicates using the following formulas:

$$(\;\;{\hat{i}}_{n},\;\;{\hat{j}}_{n})=\arg\underset{i,j}{\min}|M_{n,i}-M_{n,j}|,$$

where *i*, *j* ∈ {1, 2, 3} represents the two closest replicates from the three replicates, and *n* ∈ {1, 2, …, *N*} denotes the CGI. Then the weighted average methylation *BM*_*n*_ for CGI region *n* was computed following the previously described procedure. And the corresponding total coverage *Total*_*n*_ was given by

$$Total_{n}=Total_{n,i}+\;Total_{n,j}.$$

Additionally, we calculated the weighted average methylation ($Tumor\_BM_{n}$, $Tissue\_BM_{n}$) and the corresponding total coverage ($Tumor\_Total_{n}$ and $Tissue\_Total_{n}$,) for both breast tumor and paired tissue. For a specific CGI *n*, the mean weighted average methylation $Mean\_BM_{n}$ of tumor and tissue was determined by

$$\begin{array}{rl} & Mean\_BM_{n}=\\ & \frac{Tumor\_BM_{n}\;*\;Tumor\_Total_{n}+Tissue\_BM_{n}\;*\;Tissue\_Total_{n}}{Tumor\_Total_{n}+Tissue\_Total_{n}}. \end{array}$$

Afterward, we calculated the *Z*-score to assess whether the difference in methylation levels between tumor and tissue exceeded 10% (0.1), as indicated by the formulas

$$\begin{array}{rl} & z_{>}=\\ & \frac{Tumor\_BM_{n}-(Tissue\_BM_{n}+0.1)}{\sqrt{Mean\_BM_{n}\;*\;(1-Mean\_BM_{n})\;*\;\left(\frac{1}{Tumor\_Total_{n}}+\frac{1}{Tissue\_Total_{n}}\right)}} \end{array}$$

and

$$\begin{array}{rl} & z_{<}=\\ & \frac{Tumor\_BM_{n}-(Tissue\_BM_{n}-0.1)}{\sqrt{Mean\_BM_{n}\;*\;(1-Mean\_BM_{n})\;*\;\left(\frac{1}{Tumor\_Total_{n}}+\frac{1}{Tissue\_Total_{n}}\right)}}, \end{array}$$

where *z*_>_ represents the condition in which the methylation of CGI in the tumor is significantly greater than in the tissue by 10%, and *z*_<_ represents the condition in which the methylation of CGI in the tumor is significantly less than in the tissue by 10%. The corresponding probability is calculated as follows:

$$P_{>\;}=\;1-\phi (z_{>})$$

and

$$P_{<\;}=\;\phi (z_{<}),$$

where ϕ represents the cumulative distribution function (CDF) of the normal distribution. Therefore, the one-sided *P*-value is the minimum value between *P*_>_ and *P*_<_.

Second, we identified differentially methylated CGIs using EM-seq results. EM-seq was conducted for breast tumor and paired nontumor breast tissue with two replicates. The differential methylation analysis for CGIs was performed using edgeR (version 3.38.4) (Robinson et al. 2010) with a false discovery rate (FDR) threshold of <0.05.

Finally, we conducted a comparison between differentially methylated CGIs defined by RIMS-seq2 and EM-seq. Specifically, we focused on 1000 CGI regions with a difference in methylation levels >10% based on EM-seq results, which also had corresponding RIMS-seq2 results for comparison. True positives (TPs) were defined as CGIs identified as differentially methylated by both RIMS-seq2 and EM-seq. False positives were CGIs identified as differentially methylated by RIMS-seq2 but not by EM-seq. False negatives were CGIs identified as differentially methylated by EM-seq but not by RIMS-seq2. True negatives were CGIs identified as not differentially methylated by both EM-seq and RIMS-seq2.

### Variant calling and SNP comparison

The variant calling and comparison were performed as described previously (Zhou et al. 2019; Yan et al. 2022), except for the use of GATK version 4.2.5.0. For RIMS-seq2 WGS and TES, we applied an additional filter, “DP < 5,” to remove SNPs with low coverage. The resulting SNP sites were used in the RIMS-seq2 methylation prediction process. We used the WGS of NA12878 (generated by JIMB NIST Genome in a Bottle; JIMB WGS HG001) and K562 (ENCODE, ENCSR053AXS) (Zhou et al. 2019) as a benchmark for variant calling comparison. We restricted the comparison to the variants on somatic chromosomes and Chr X.

## Data access

All raw and processed sequencing data generated in this study have been submitted to the NCBI Gene Expression Omnibus (GEO; https://www.ncbi.nlm.nih.gov/geo/) under accession number GSE234235. The RIMS-seq code is available at GitHub (https://github.com/elitaone/RIMS-seq2) and as Supplemental Code.

## Supplementary Material

Supplement 1

Supplement 2

Supplement 3

Supplement 4

Supplement 5

Supplement 6

Supplement 7
