# Supplementary material for: Simultaneous assessment of human genome and methylome data in a single experiment using limited deamination of methylated cytosine
Source: Genome Res. 2024 Jun;34(6):904–13. doi: 10.1101/gr.278294.123 (PMC11293541; doi:10.1101/gr.278294.123)
Supplement: Supplement 6 [file Supplemental_Figures.pdf]

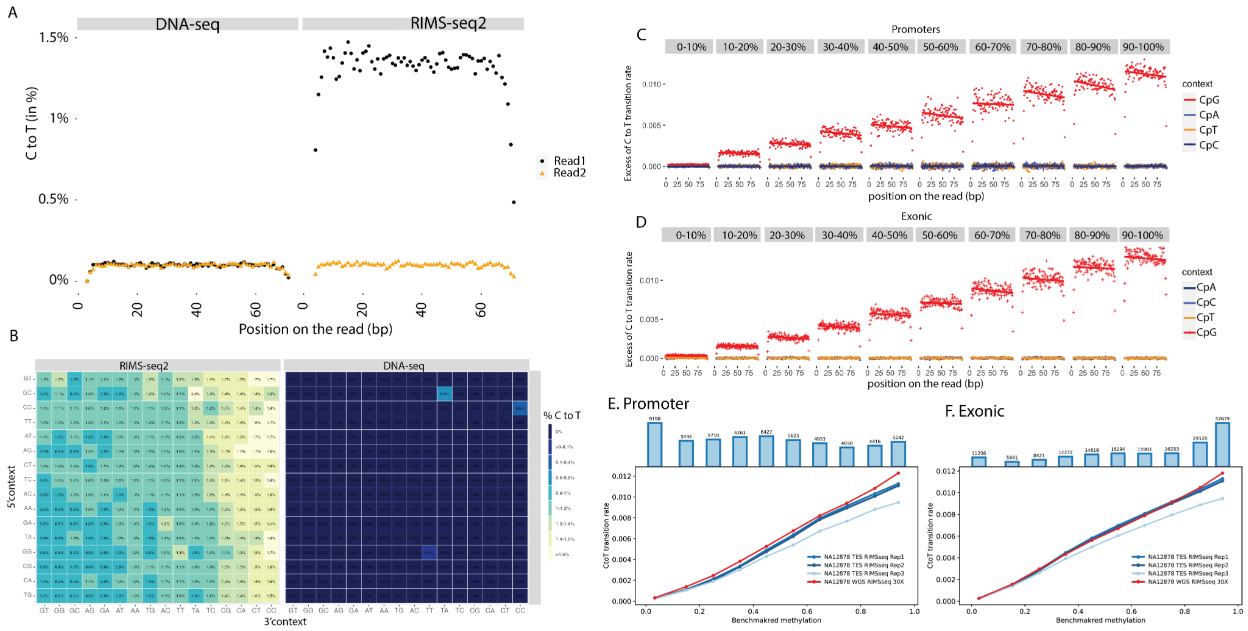

Supplemental Fig. S1: DNA-seq and RIMS-seq2 performed on Enterobacteria phage T4gt (5hmC): **(A)** C to T transition using DNA-seq (left panel) or RIMS-seq2 (right panel) for paired-end read 1 (black) and read 2 (yellow) function of the position on the read. **(B)** C to T transition using DNA-seq (right panel) or RIMS-seq2 (left panel) function of the sequence context. Whole human genome RIMS-seq2 excess of C to T transition rates for **(C)** Promoter regions and **(D)** exonic regions binned into 1-10% to 9-100% methylation levels function of the position on the read. The rate of C to T transition was computed for CpG, CpA, CpT and CpC contexts. The rate of C to T transition and benchmarked methylation fit a positive linear regression model for **(E)** Promoter regions and **(F)** exonic regions in Whole genome RIMS-seq2 (red line) and all targeted RIMS-seq2 (blue lines). Blue bar plots represent the number of genomic regions for each binned methylation level.

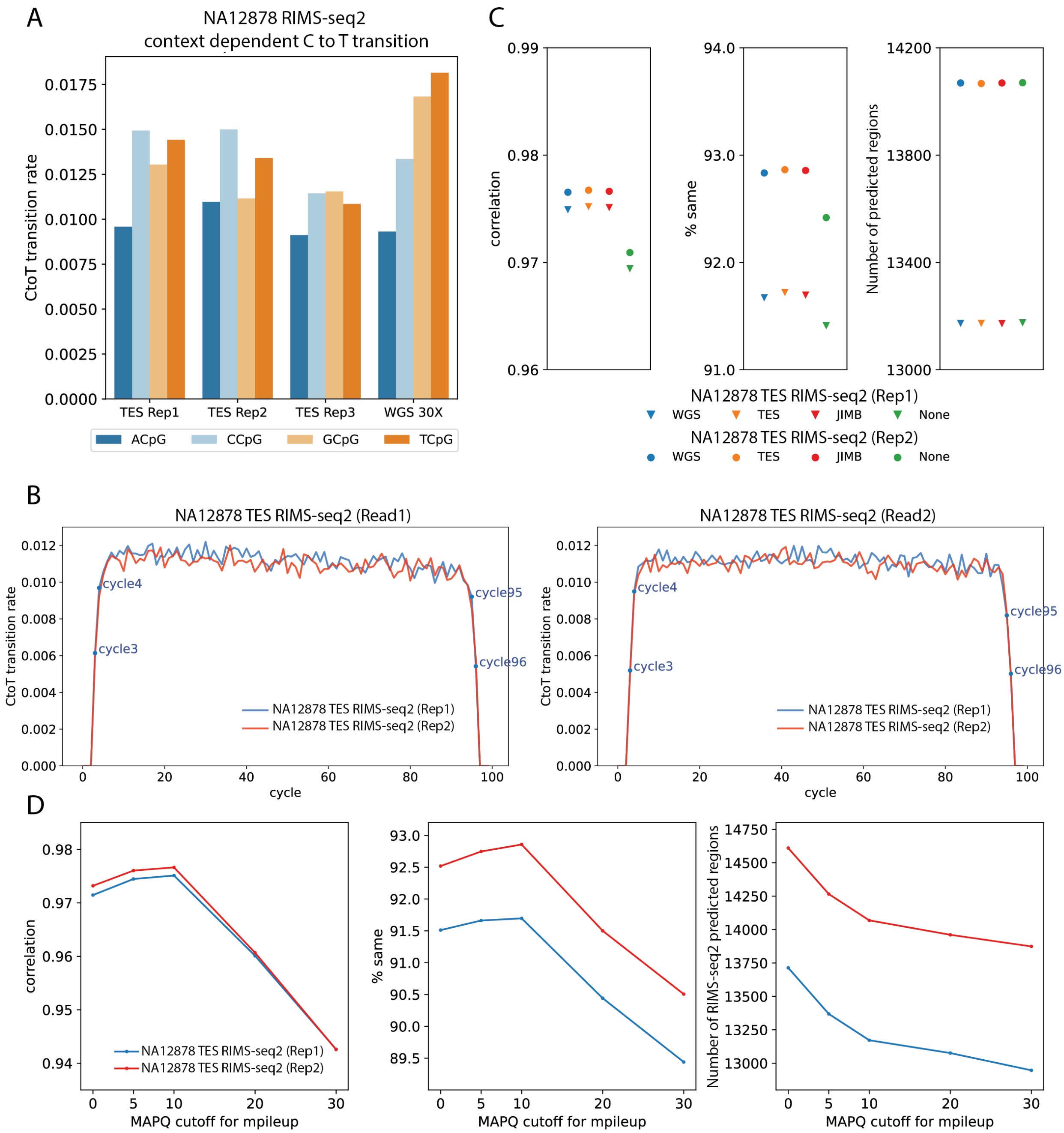

Supplemental Fig. S2 : Additional parameters affecting calibration. **A**. Sequence context : C to T transition rates in the stably hypermethylated regions for the ApCpG (blue), CpCpG (light blue), GpCpG (light orange) and TpCpG (orange) sequence context for the three RIMS-seq2 exome sequencing replicates (TES Rep1, 2 and 3) and whole genome sequencing downsampled to 30 fold coverage (WGS 30x) **B**. sequencing cycle : C to T transition rates in the stably hypermethylated regions function of the sequencing cycles for two exome replicates for the first (left) and second (right) paired end read. **C.** SNP: Confounding effect of germline variation on methylation calls **D.** MAPQ cutoff : effect of MAPQ cutoff on methylation calls.

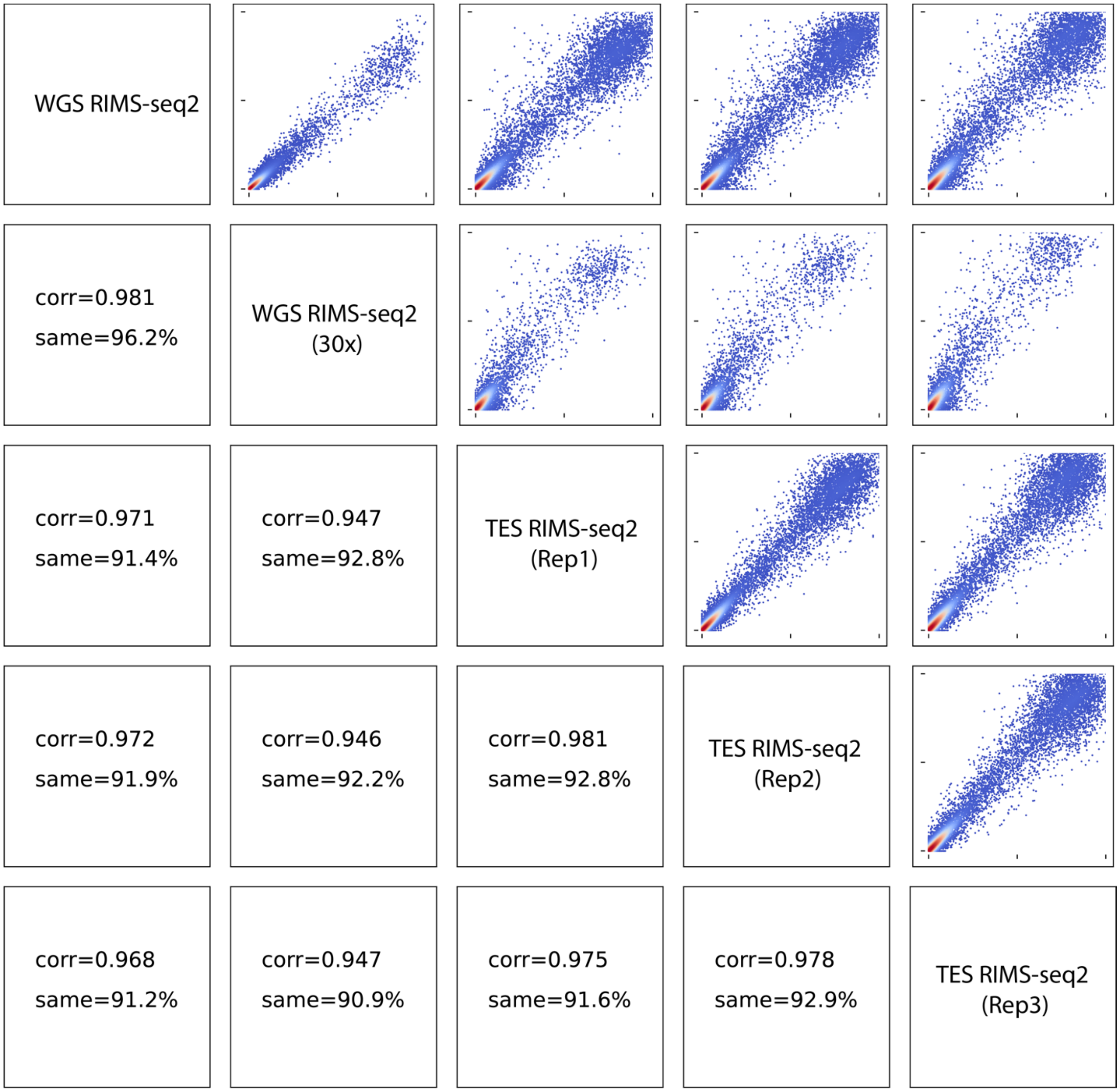

Supplemental Fig. S3 : RIMS-seq2 replicate correlations. Comparison of the methylation levels of CGI (RAML) for one whole genome sequencing (WGS, full dataset and downsampled to 30x coverage) and exome sequencing performed in triplicates (TES Rep1, Rep2 and Rep3). Corr stands for Pearson correlation. Same represents the percent of CGI that are quantified in the same methylation category (high, medium or low) by both methods (Figure 2B has a representation of the quadrants).

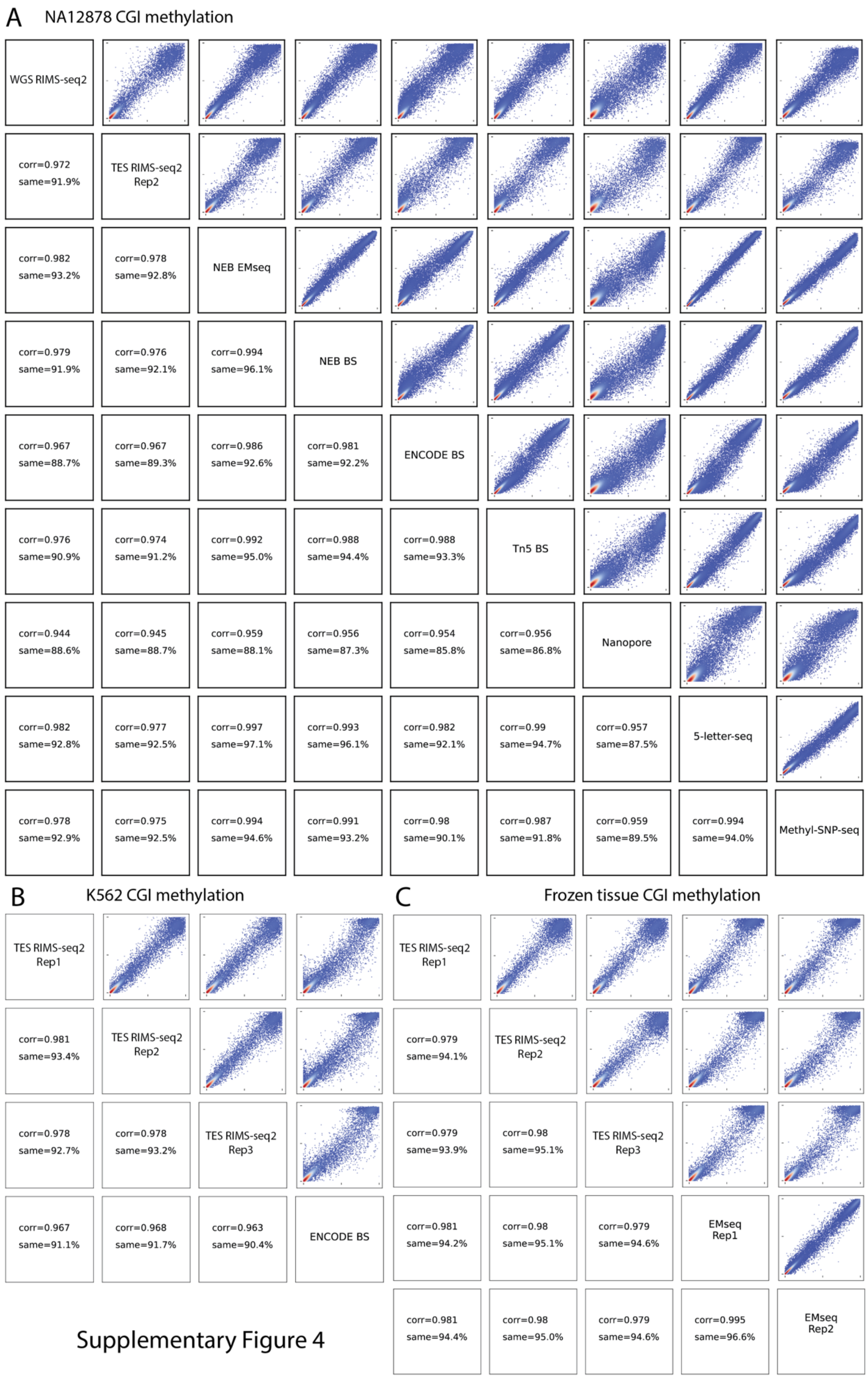

Supplementary Figure 4

Supplemental Fig. S4: RIMS-seq2 correlations with other sequencing technologies. CGI methylation quantification of **A** NA12878 **B.** K562 **C.** Frozen tissue. BS: bisulfite sequencing. Tn5 BS: Bisulfite-tagging sequencing. Corr stands for Pearson correlation. Same represents the percent of CGI that are quantified in the same methylation category (high, medium or low) by both methods.

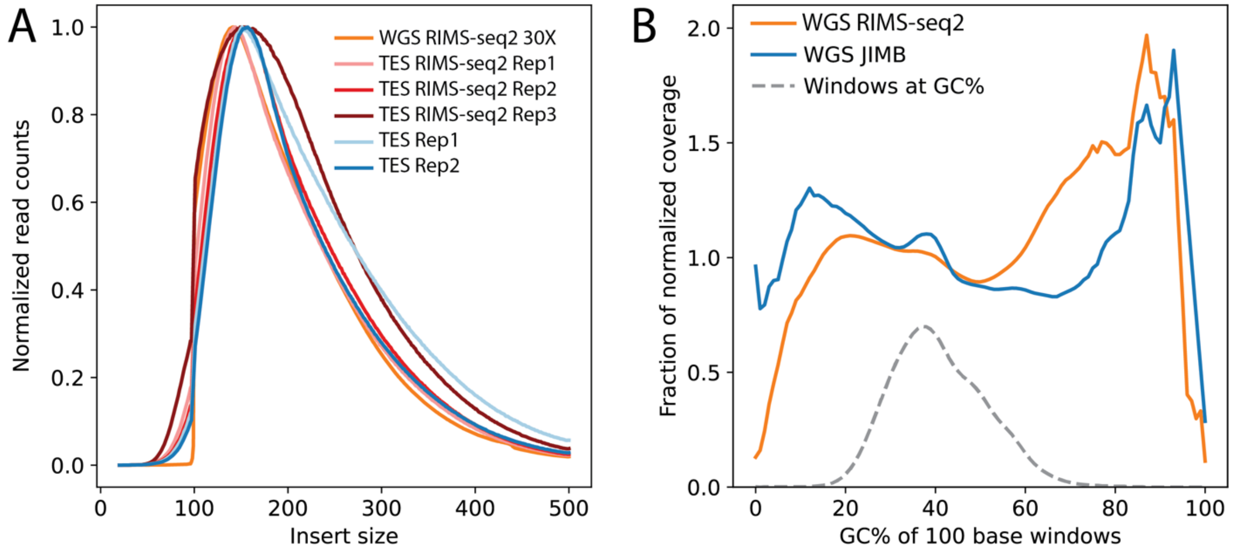

Supplemental Fig. S5 : Standard quality control for DNA-seq. A. Insert size distribution (in bp) for RIMS-seq2 libraries (orange for WGS, light red, red and dark red for TES) and TES DNA sequencing (blue and light blue). **B.** CG bias for the WGS RIMS-seq2 (orange) and WGS DNA-seq (blue) whole genome sequencing.
